# Depressive effectiveness of vigabatrin (γ-vinyl-GABA), an antiepileptic drug, in intermediate-conductance calcium-activated potassium channels in human glioma cells

**DOI:** 10.1186/s40360-021-00472-3

**Published:** 2021-01-13

**Authors:** Te-Yu Hung, Huai-Ying Ingrid Huang, Sheng-Nan Wu, Chin-Wei Huang

**Affiliations:** 1 Department of Pediatrics, Chi-Mei Medical Center, Tainan, Taiwan; 2 Neuroscience Program, McGill University, Montréal, Quebec Canada; 3 Department of Physiology, College of Medicine, National Cheng Kung University, No. 1, University Road, Tainan City, 70101 Taiwan; 4 Institute of Basic Medical Sciences, National Cheng Kung University Medical College, Tainan City, Taiwan; 5 Department of Medical Research, China Medical University Hospital, China Medical University, Taichung City, Taiwan; 6 Department of Neurology, National Cheng Kung University Hospital, College of Medicine, National Cheng Kung University, No. 1, University Road, Tainan City, 70101 Taiwan

**Keywords:** Vigabatrin, Intermediate-conductance Ca^2+^-activated K^+^ channel, Glioma cell

## Abstract

**Background:**

Vigabatrin (VGB) is an approved non-traditional antiepileptic drug that has been revealed to have potential for treating brain tumors; however, its effect on ionic channels in glioma cells remains largely unclear.

**Methods:**

With the aid of patch-clamp technology, we investigated the effects of VGB on various ionic currents in the glioblastoma multiforme cell line 13–06-MG.

**Results:**

In cell-attached configuration, VGB concentration-dependently reduced the activity of intermediate-conductance Ca^2+^-activated K^+^ (IK_Ca_) channels, while DCEBIO (5,6-dichloro-1-ethyl-1,3-dihydro-2H-benzimidazol-2-one) counteracted the VGB-induced inhibition of IK_Ca_ channels. However, the activity of neither large-conductance Ca^2+^-activated (BK_Ca_) nor inwardly rectifying K^+^ (K_IR_) channels were affected by the presence of VGB in human 13–06-MG cells. However, in the continued presence of VGB, the addition of GAL-021 or BaCl_2_ effectively suppressed BK_Ca_ and K_IR_ channels.

**Conclusions:**

The inhibitory effect of VGB on IK_Ca_ channels demonstrated in the current study could be an important underlying mechanism of VGB-induced antineoplastic (e.g., anti-glioma) actions.

## Background

Vigabatrin (VGB; γ-vinyl-gamma-aminobutyric acid [γ-vinyl-GABA]) is an approved antiepileptic drug, which is tailored as an adjuvant therapy for adults with refractory partial epilepsy; it is also used for the treatment of infantile spasms [1–3]. VGB is a structural analog of GABA, which irreversibly inhibits GABA-transaminase [4] and thus consequently increases levels of the inhibitory neurotransmitter GABA [5] in the brain. It has been shown to attenuate astroglial TWIK-related acid-sensitive K^+^ channel-1 in the hippocampus of seizure-sensitive gerbils [6]. Although most of VGB’s effects are thought to be largely attributed to its GABA-ergic actions, its perturbations on the amplitude or gating of ionic effects are not clear.

The degree of functional expression in the intermediate-conductance Ca^2+^-activated K^+^ (IK_Ca_) channels identified in glioma cells has recently been disclosed to interfere with the progression of malignant tumors [7]. IK_Ca_ channels (also known as K_Ca_3.1, SK4, IK_Ca_1, or KCNN4) are encoded by the *KCNN4* gene. These channels have been cloned from human, mouse, or rat tissues; and, their activities are viewed to be associated with various cellular functions, which include hormonal secretion, cell motility or proliferation, and the regulation of Ca^2+^ influx or K^+^ efflux. All of these underlying mechanisms have been extensively studied in different types of non-excitable or neoplastic cells [8–10]. Alternatively, these channels have single-channel conductance of 20–60 pS and their biophysical and pharmacological profiles are viewed to be distinguishable from those of large- or small-conductance Ca^2+^-activated K^+^ channels [11, 12]. Of importance, the modulators of IK_Ca_ channels represent a potential therapeutic approach for a variety of diseases, particularly at malignant gliomas [7, 13].

VGB has been reported to decrease oligodendrocyte precursor cell proliferation as well as to increase the number of mature oligodendrocytes [14]. Interestingly, it has been also disclosed to have promising therapeutic efficacy for treating brain metastases in vivo [15]. However, the ionic mechanism through which VGB exerts anti-neoplastic actions is not yet determined. In this study, we sought to investigate its ionic mechanism which could be linked to anti-neoplastic actions in the glioblastoma multiforme cell line (i.e., human 13–06-MG glioma cells).

## Methods

### Chemicals, drugs and solutions

VGB ((±)-γ-vinyl-GABA, C_6_H_11_NO_2_) was acquired from Sigma-Aldrich (Merck Ltd., Taipei, Taiwan), GAL-021 was from MedChemExpress (Everything Biotech Ltd., New Taipei City, Taiwan), while DCEBIO (5,6-dichloro-1-ethyl-1,3-dihydro-2*H*-benzimidazol-2-one) and TRAM-34 (1-((2-chlorophenyl)-(diphenyl)methyl)-1*H*-pyrazole) were from Tocris (Union Biomed, Taipei, Taiwan). Unless stated otherwise, for cell preparations, all culture media, fetal bovine serum, L-glutamine, and trypsin/EDTA were acquired from HyClone™ (Thermo Fisher; Level Biotech, Tainan, Taiwan); and, all other chemicals or reagents were of analytical grade.

The composition of the bathing solution (i.e., HEPES-buffered normal Tyrode’s solution) was 136.5 mM NaCl, 5.4 mM KCl, 1.8 mM CaCl_2_, 0.53 mM MgCl_2_, 5.5 mM glucose, and 5.5 mM HEPES adjusted with NaOH to pH 7.4. To measure K^+^ currents, we backfilled the patch pipettes with an internal solution consisting of 130 mM K-aspartate, 20 mM KCl, 1 mM KH_2_PO_4_, 1 mM MgCl_2_, 3 mM Na_2_ATP, 100 μM Na_2_GTP, 0.1 mM EGTA, and 5 mM HEPES adjusted with KOH to pH 7.2 [16, 17]. To avoid the contamination of whole-cell Cl^−^ currents, we substituted Cl^−^ ions inside the pipette solution for aspartate.

For recording large-conductance Ca^2+^-activated (BK_Ca_) channels, we kept cells in a high K^+^-bathing solution, and its composition was 145 mM KCl, 0.53 mM MgCl_2_, and 5 mM HEPES adjusted with KOH to 7.2, and the pipette solution contained 145 mM KCl, 2 mM MgCl_2_, and 5 mM HEPES titrated with KOH to 7.2. In this study, we obtained the reagent water from a Milli-Q water purification system (Merck, Ltd., Taipei, Taiwan). The culture medium and pipette solution were filtered on the day of use with an Acrodisc® syringe filter with a Supor® membrane (Bio-Check; New Taipei City, Taiwan).

### Cell preparations

The glioblastoma multiforme cell line (13–06-MG) used in this study was kindly provided by Professor Dr. Carol A. Kruse (Department of Neurosurgery, Ronald Reagan UCLA Medical Center, LA, U.S.A). The 13–06-MG cells were cultured at a density of 10^6^/ml in high glucose (4 g/l) Dulbecco’s modified Eagle media (Invitrogen, Carlsbad, CA, USA) supplemented with 10% heat-inactivated fetal bovine serum, 100 U/ml penicillin and 10 μg/ml streptomycin. Cells were maintained at 37 °C in a 5% CO_2_ incubator as monolayer cultures and thereafter sub-cultured weekly. Fresh media was added every 2–3 days in order to ensure a healthy cell population. To verify the presence of glial cells, we identified them by displaying glial fibrillary acidic protein, which is a cytoskeletal protein.

To evaluate concentration-dependent inhibition of VGB on the probability of IK_Ca_ channels that would be open, we kept 13–06-MG cells to be bathed in normal Tyrode’s solution containing 1.8 mM CaCl_2_, and each cell examined was voltage-clamped at − 80 mV relative to the bath. The probability of channel opening was measured in the control or during cell exposure to different concentrations (0.3–100 μM) of VGB; and, these values were then compared with those taken after the addition of TRAM-34 (3 μM). TRAM-34 is a known selective blocker of IK_Ca_ channels. The concentration required to suppress 50% of channel activity was determined by means of a Hill function:

$$ Percentageinhibition=\frac{E_{\mathrm{max}}\times {\left[C\right]}^{n_H}}{IC_{50}^{n_H}+{\left[C\right]}^{n_H}}, $$

where IC_50_ or *n*_H_ is the concentration required for a 50% inhibition or the Hill coefficient, respectively; [C] the VGB concentration; and *E*_max_ the maximal reduction in channel opening probability (i.e., TRAM-34-sensitive channel activity) caused by VGB.

### Statistical analyses

Linear or nonlinear curve-fitting (e.g., sigmoidal or exponential curve) to the data sets collected was performed by using either Microsoft Excel® (Redmond, WA) or OriginPro 2016 (Microcal). The experimental data are presented as the mean ± standard error of the mean (SEM) with sample sizes (n) indicating the number of 13–06-MG cells from which the results was acquired. The Student’s *t*-test (paired or unpaired) or one-way analysis of variance (ANOVA) followed by a *post-hoc* Fisher’s least-significant difference test, was performed to analyze multiple groups. The data were examined using a nonparametric Kruskal-Wallis test, subject to possible violation in the normality underlying ANOVA. Differences were considered statistically significant when the *P*-value was below 0.05.

## Results

### *VGB* and the activity of IK_Ca_ channels in 13–06-MG cells

**Fig. 1:**
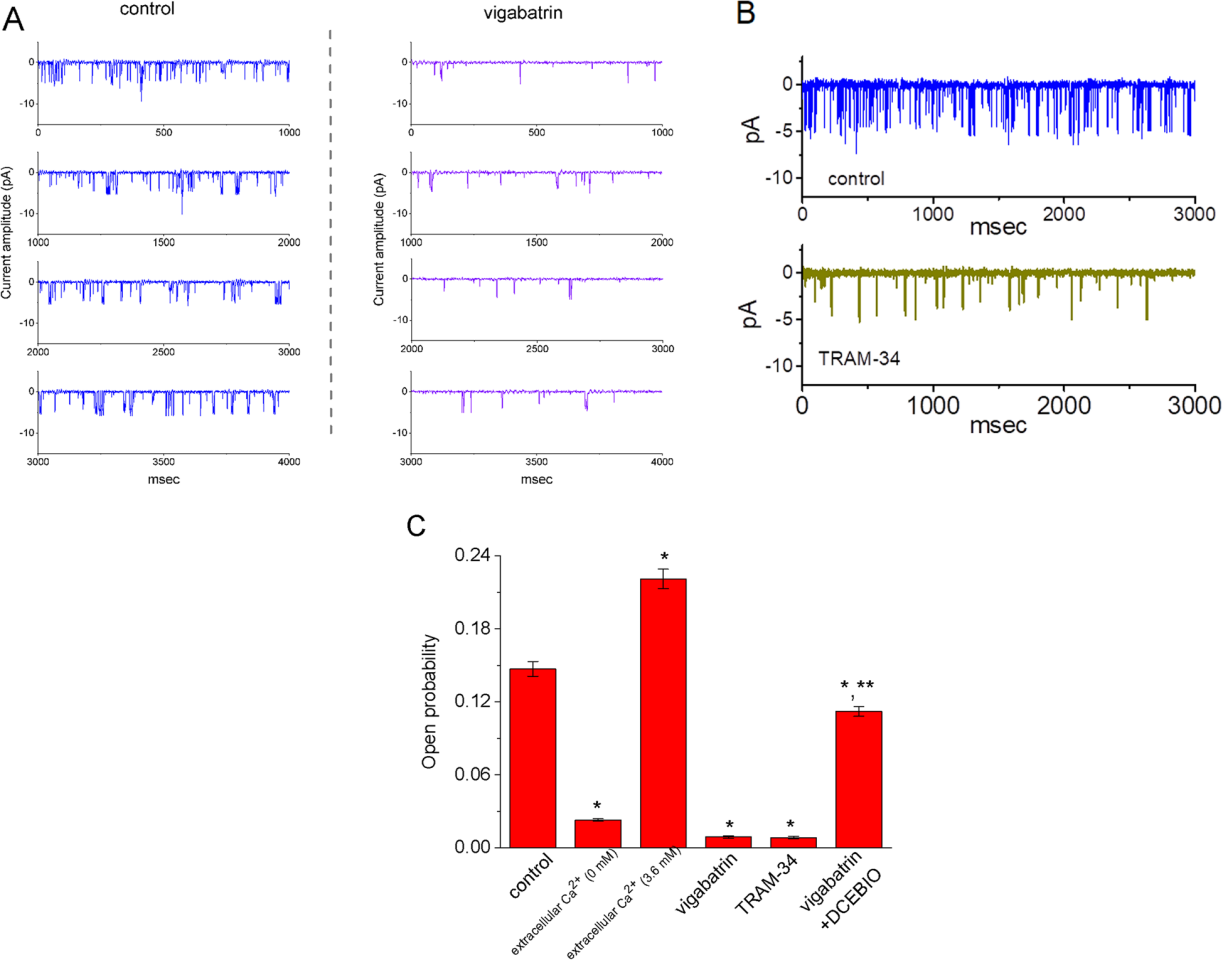
Effect of VGB on the activity of intermediate-conductance Ca^2+^-activated K^+^ (IK_Ca_) channels expressed in human 13–06-MG glioma cells. In this set of experiments, 13–06-MG cells were bathed in normal Tyrode’s solution containing 1.8 mM CaCl_2_ and single-channel current recordings were made. The probability of IK_Ca_ channels opening was measured at − 80 mV relative to the bath. **a** Original current traces for IK_Ca_ channels obtained in the absence (left) and presence (right) of VGB (10 μM). Note that channel opening gives a downward deflection in current. **b** Original IK_Ca_ channel traces taken in the absence (upper) and presence (lower) of TRAM-34 (3 μM). **c** Summary of the data showing the effects of control, extracellular Ca^2+^ (0 mM), extracellular Ca^2+^ (3.6 mM), VGB, TRAM-34 (3 μM), and VGB (10 μM) plus DCEBIO (10 μM) on IK_Ca_ channel activity. The probability of IK_Ca_ channel opening was measured at − 80 mV relative to the bath. Each bar indicates the mean ± SEM (*n*=9–11). ^*^Significantly different from control (i.e., in the presence of 1.8 mM Ca^2+^, but VGB was not present) (*P*< 0.05) and ^**^significantly different from the VGB alone group (*P*< 0.05)

Experiments to evaluate the effect of VGB on IK_Ca_ channel activity were performed. In this set of experiments, 13–06-MG cells were bathed in normal Tyrode’s solution containing 1.8 mM CaCl_2_ and single-channel current recordings were made. The probability of IK_Ca_ channel opening was measured at − 80 mV relative to the bath. In the presence of VGB, the IK_Ca_ channels were significantly less likely to be open, compared with the control (Fig. 1a). Similar effects were observed after TRAM-34 was added to the control group (Fig. 1b). IK_Ca_ channels that were closed in VGB-treated cells were reopened after the cells were treated with DCEBIO, an activator of IK_Ca_ channels. This data is summarized in Fig. 1c, which shows the effects of control, extracellular Ca^2+^ (0 mM), extracellular Ca^2+^ (3.6 mM), VGB, TRAM-34 (3 μM), and VGB (10 μM) plus DCEBIO (10 μM) on IK_Ca_ channel activity. Each bar indicates the mean ± SEM (*n*=9–11). As cells were exposed to Tyrode’s solution containing 3.6 mM CaCl_2_, the presence of VGB (10 μM) effectively decreased IK_Ca_ channel activity, while it had minimal effect on it in cells bathed in Ca^2+^-free Tyrode solution. Therefore, the results enable us to indicate that the IK_Ca_ channels measured from these cells was sensitive either to the level of extracellular Ca^2+^ or to block by TRAM-34, and that VGG-mediated inhibition of IK_Ca_ channel was attenuated by further application of DCEBIO.

### *VGB* effect on single-channel conductance of IK_Ca_ channels

**Fig. 2:**
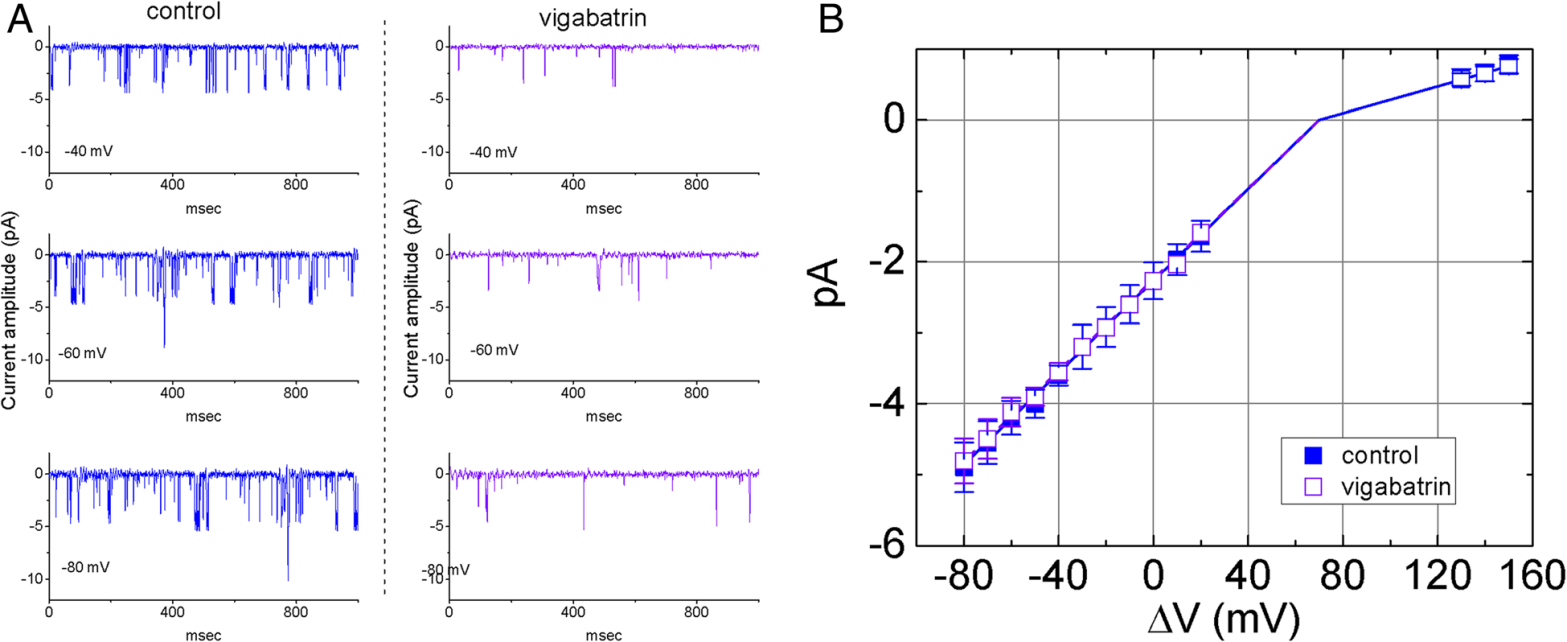
Original current traces of single channel activities and the association between single IK_Ca_ channel amplitude and membrane potential obtained in the absence (left) and presence of VGB (10 μM). **a** Original IK_Ca_ channel currents obtained with or without the addition of VGB (10 μM). The number shown at the left lower corner of each panel indicates the level of membrane potential relative to the bath. The downward deflection is the opening event of the channel. **b** The association between single IK_Ca_ channel amplitude and membrane potential (i.e., Δvoltage) in the absence (■) and presence (□) of 10 μM VGB (mean ± SEM; *n*=8–13 for each point). Note that the single-channel conductance of IK_Ca_ channels over the voltage range between − 80 and − 40 mV obtained in the absence (32.4 pS) and presence (32.1 pS) of VGB did not differ significantly in human 13–06-MG cells

How VGB treatment affected IK_Ca_ channels at different membrane potentials was further evaluated. Plots of current amplitude as a function of holding potential were then constructed. Single-channel amplitudes at the potentials ranging between − 80 and + 20 mV were measured. Original current traces of single channel activities at the different levels of membrane potential relative to the bath obtained in the absence (left) and presence (right) of VGB (10 μM) were shown (Fig. 2a). The single-channel conductance of IK_Ca_ channels calculated from a linear *I-V* relationship in the control was further calculated to yield 32.4±4 pS (*n*=9) over the voltage ranging between − 80 and + 20 mV (Fig. 2b). Of notice, the conductance measured at negative potentials was greater than that at positive voltages. However, the single-channel slope conductance (32.1±4 pS; *n*=9, *P*> 0.05) of IK_Ca_ channels was not significantly changed after VGB (10 μM) treatment, despite the observed reduction in the probability of channel openings.

### Concentration-dependent inhibitory effect of *VGB* on the activity of IK_Ca_ channels

**Fig. 3:**
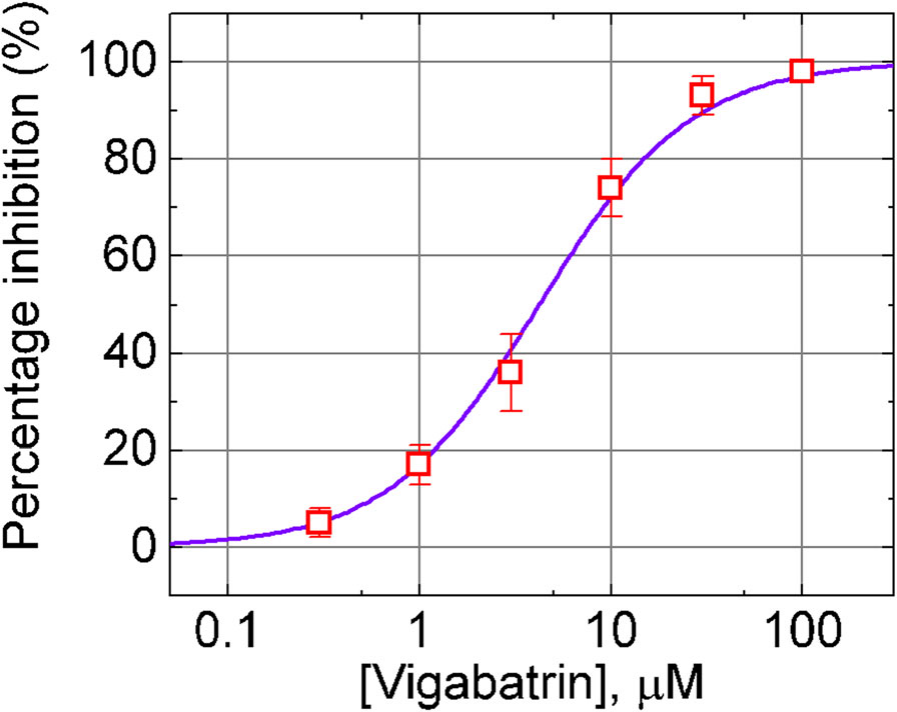
Concentration-response curve for VGB-induced suppression of IK_Ca_ channels recorded in human 13–06-MG cells (mean ± SEM; *n*=11–14 for each point). VGB was added at various concentrations (0.3–100 μM) to the bath, and the activity of IK_Ca_ channels was detected at − 80 mV relative to the bath. The smooth curve was well fitted with a least-squares procedure to a modified Hill function

The relationship of the percentage suppression of IK_Ca_ channel activity versus VGB concentration was further analyzed. In this set of experiments, each cell was maintained at --80 mV relative to the bath, and the channel open-state probabilities in the absence and presence of different VGB concentrations were measured. As depicted in Fig. 3, the addition of VGB (0.3–100 μM) suppressed the activity of IK_Ca_ channels in a concentration-dependent manner. The IC_50_ value required for its inhibitory effect on channel activity in 13–06-MG cells was calculated to be 4.21 μM, and it at a concentration of 100 μM nearly abolished the probability of channel openings. Findings from these observations led us to indicate that VGB is able to exert a depressive action on the activity of IK_Ca_ channels expressed in 13–06-MG cells.

### Effect of VGB and VGB plus GAL-021 on the probability of BK_Ca_ channel opening

**Fig. 4:**
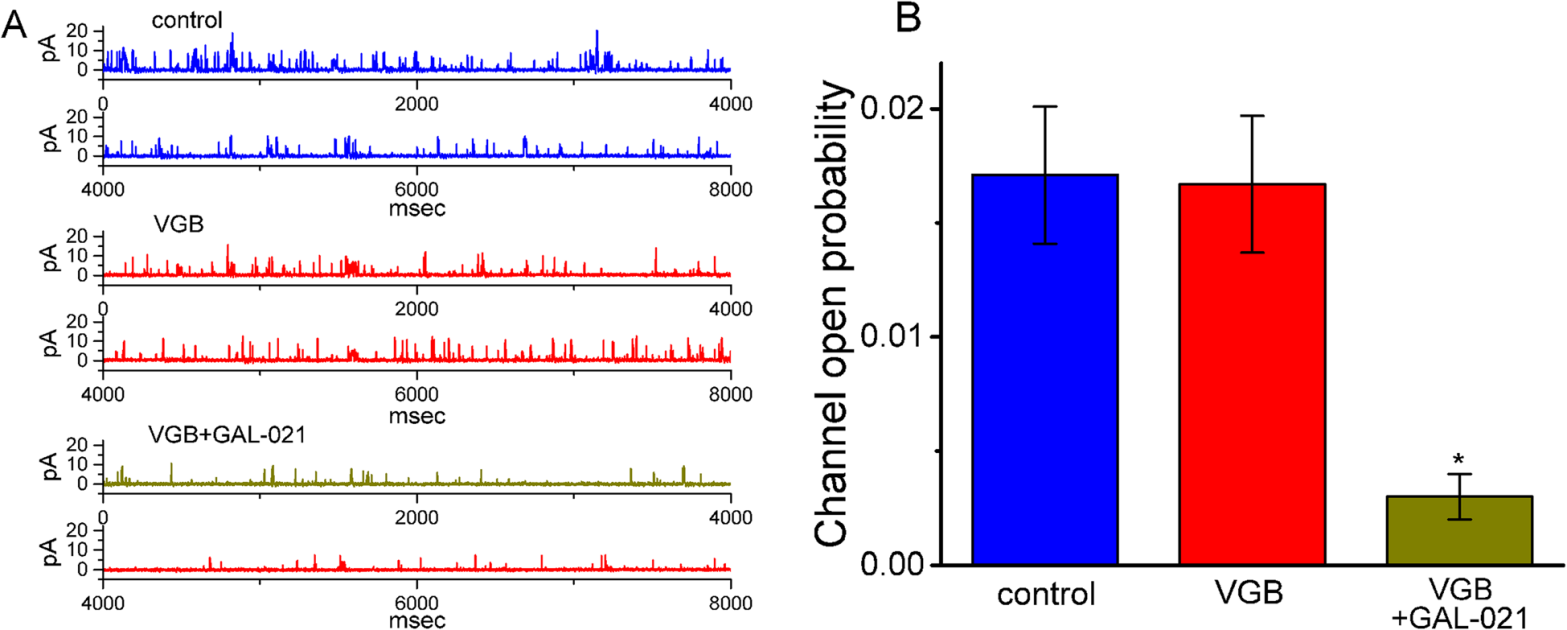
The inability of VGB to alter the activity of BK_Ca_ channels was recorded in human 13–06-MG cells. The experiments were conducted under cell-attached configuration. The cells were bathed in a high-K^+^ solution containing 1.8 mM CaCl_2_, and the examined cells were clamped at a level of + 80 mV. **a** Original trace of single BK_Ca_ channels obtained in the control (upper) and after the addition of 10 μM VGB (middle) or 10 μM VGB plus 10 μM GAL-021 (lower). The upward deflection indicates the opening event of the channel. **b** Summary bar graph of the effects of VGB or VGB plus GAL-021 on the probability of BK_Ca_ channel opening (mean ± SEM; *n*=7 for each bar). ^*^Significantly different from the control or 10 μM VGB alone (*P*< 0.05)

We further examined whether the presence of VGB could affect the activity of BK_Ca_ channels in 13–06-MG cells. In these experiments, cells were immersed in a high-K^+^ solution that contained 1.8 mM CaCl_2_, and the examined cells were held at + 80 mV. As the cells were exposed to 10 μM VGB, the probability of BK_Ca_ channels opening was not altered (Fig. 4). However, following the addition of GAL-021 (10 μM) channel activity was significantly decreased. GAL-021 has been previously reported to be a blocker of BK_Ca_ channels [18]. Unlike IK_Ca_ channels, which were suppressed by VGB, the BK_Ca_ channels were resistant to being blocked by this agent.

### Effect of VGB and VGB plus BaCl_2_ on K_IR_ channel activity

**Fig. 5:**
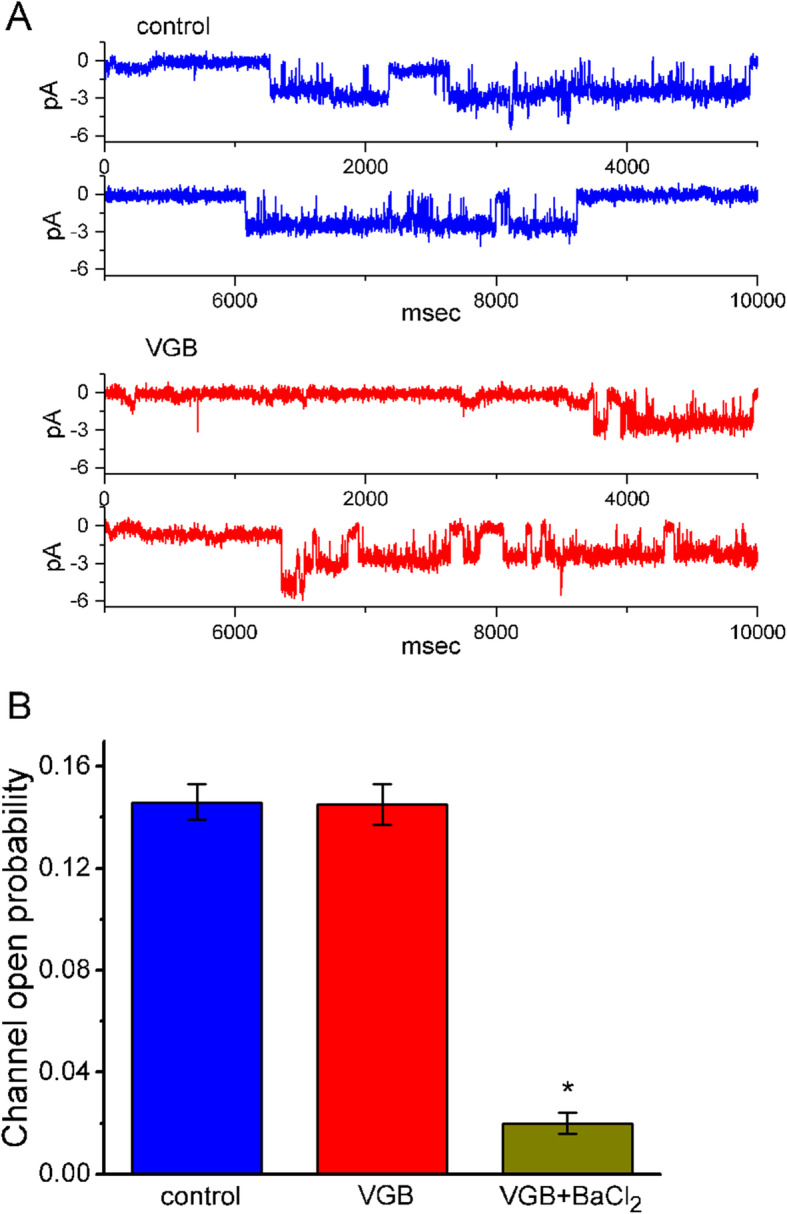
Failure of VGB to modify the activity of K_IR_ channels in human 13–06-MG cells. In this set of experiments, we bathed cells in Ca^2+^-free Tyrode’s solution and, during the recording, we backfilled the pipette by using K^+^-containing solution. The activity of K_IR_ channels was detected at − 80 mV relative to the bath. **a** Single K_IR_ channels obtained in the absence (upper) and presence (lower) of 10 μM VGB. The downward deflection denotes the channel opening event. **b** Summary bar graph depicting the effect of VGB and VGB plus BaCl_2_ on the activity of K_IR_ channels in human 13–06-MG cells (mean ± SEM; *n*=7 for each bar). ^*^Significantly different from the control or 10 μM VGB alone (*P*< 0.05). VGB: 10 μM VGB; BaCl_2_: 1 mM BaCl_2_

In another set of single-channel current recordings, we tested whether other K^+^ channels (i.e., K_IR_ channels) could be affected by the presence of VGB. Cells were bathed in Ca^2+^-free Tyrode’s solution and the holding potential was set at − 80 mV relative to the bath. However, the presence of 10 μM VGB was unable to produce any modifications in K_IR_ channel activity in these cells (Fig. 5). However, the subsequent addition of 1 mM BaCl_2_ in the continued presence of 10 μM VGB, effectively suppressed the probability of channel opening. BaCl_2_ is regarded as an inhibitor of K_IR_ channels [19].

## Discussion

VGB is an anti-epileptic agent that is viewed to be an inhibitor of gamma-aminobutyric acid (GABA) breakdown. It has been approved for use as an adjunctive treatment for resistant epilepsy, and as a monotherapy for infantile spasms or West syndrome [2, 3]. In the present study, we found that VGB dose-dependently lessened the probability of IK_Ca_-channel openings, and that this reduction in channel activity is voltage-dependent and associated with a rise in mean closed time of the channel. The reduction in the channel open-state probability accounts primarily for its suppression in IK_Ca_ channel activity, owing to the inability to modify single-channel conductance of the channel. However, the activity of neither BK_ca_ nor K_IR_ channels was conceivably perturbed by the presence of VGB. Therefore, in addition to its inhibition of GABA breakdown, this study revealed that VGB suppressed the activity of IK_Ca_ channels. This effect could be partly responsible for its suppression of neoplastic cells [20]. Therefore, awareness needs to be appropriately made when the effect of this compound is explained solely by its action on GABA-ergic dysregulation [14]. However, whether there is functional coupling between GABA-receptor(s) signaling and IK_Ca_ channel activity remains to be further studied.

The single-channel conductance of IK_Ca_ channels in human glioma cells (13–06-MG) was calculated to be 32 pS, a value similar to that of the prototypical IK_Ca_ channels present in other cell types [7, 13, 21], but less than that of BK_Ca_ channels [22, 23]. VGB-mediated inhibition of IK_Ca_ channel activity depends on membrane voltage and it is viewed to occur via a direct interaction with the K_Ca_3.1 channel protein in glioma cells.

In this study, the IC_50_ value required for VGB-induced inhibition of IK_Ca_ channels was 4.21 μM. There is a wide range of serum/plasma concentrations (0.8–36 mg/L) associated with successful epilepsy treatment [24]. The concentration in cerebrospinal fluid was noted to be approximately 30–40% of plasma concentration, supporting that the IC_50_ value of VGB observed in this study could be of clinical or therapeutic relevance. Of note, the presence of VGB inhibits the activity of IK_Ca_ channels in humans at these relatively low concentrations, and in contrast to other compounds that disrupt the GABA neurotransmission, the VGB molecule is lipophilic and able to cross the blood-brain barrier [25]. Therefore, findings from the present observations could be important in determining VGB’s in vivo anti-neoplastic mechanism.

Different types of kinetic behaviors perturbed by VGB might facilitate its inhibition of IK_Ca_ channel activity. VGB has no discernible effect on IK_Ca_ single-channel conductance; therefore, the VGB molecule unlikely acts within the channel’s central pore. However, the mean closed time of the channel was lengthened in its presence. The activity of IK_Ca_ channels has been reported to regulate the proliferation of prostate cancer cells by controlling Ca^2+^ entry into these cells [8]. However, significant changes in neither BK_Ca_ nor K_IR_ channel activity were observed in these cells. The effectiveness of VGB in inhibiting IK_Ca_ channels demonstrated presently in glioma cells does not result secondarily from the reduction of intracellular Ca^2+^ [26]. In this study, VGB inhibited IK_Ca_ channel activity within a few minutes in 13–06-MG cells. As the onset of inhibition was rapid, its action on channel activity was unlikely to ascribe from the binding to nuclear DNAs. The mechanism through which the VGB molecule interacts with IK_Ca_ channels tends to be direct and not genomic.

An earlier study in which immunolabelling of K_Ca_3.1 channels was performed, disclosed that IK_Ca_ channels tended to be differentially expressed in excitatory and inhibitory neurons of the central nervous system [21]. Different isoforms of KCa3.1 might also be present in different tissues, including gliomas; however, whether VGB is capable of modifying different types of IK_Ca_ channels remains unknown. Further studies investigating the extent to which VGB-induced effects on glioma cells may be attributed to direct inhibitory perturbations on IK_Ca_ channels, are thus needed.

Of notice, the expression and function of glial Kir channels have been previously studied in retinal Müller glial cells, Schwann cells, astrocytes, and oligodendrocytes. Expression of Kir4.1 was identified in brain and retinal glial cells, while those of Kir2.1 and Kir2.3 were reported to be present in Schwann cells [27, 28]. Whether VGA can perturb the activity of different types of Kir channels in glial cells still remains to be further resolved.

Interestingly, one in vitro study suggested that VGB should not be used for prophylaxis or the short-term treatment of epilepsy in glioblastoma [20]. However, another report suggested that blocking GABA flux into the TCA cycle, either through genetic depletion of GAD1 or pharmacological treatment with VGB, suppressed aggressive metastatic outgrowth in the brain. Furthermore, it suggests that VGB might bring an additional benefit of stabilizing tumor-induced seizures [15].

Our previous study on temozolomide, which demonstrated its inhibitory effect on IK_Ca_ accompanied by membrane depolarization, could describe an important underlying mechanism of temozolomide-induced anti-neoplastic actions [29]. Supportively, it has been reported that ionizing radiation could stimulate BK_Ca_ channel activity, resulting in Ca^2+^/calmodulin-dependent kinaces II, leading to glioblastoma cell migration [30]. As K_Ca_3.1 has been reported to confer radioresistance to breast cancer cells [31], strategies targeting KCa3.1 in anti-cancer treatment tend to be potential in modulating anti-neoplastic activity [32].

The inhibitory effect of VGB on IK_Ca_ channels demonstrated herein sheds light on and supports the potential of VGB on antineoplastic actions. The possible link between vigabatrin/IK_Ca_ channel activity and neoplastic cell behavior, including migration, spread, survival and proliferation is worth further investigation.

## Conclusion

Our study demonstrated that the inhibitory effect of VGB on IK_Ca_ channels could be an important underlying mechanism of VGB-induced antineoplastic actions.

## Data Availability

The datasets used and/or analysed during the current study are available from the corresponding author on reasonable request.

## Abbreviations

BK_Ca_ channel: Large-conductance Ca^2+^-activated K^+^ channel
DCEBIO: 5,6-dichloro-1-ethyl-1,3-dihydro-2*H*-benzimidazol-2-one
IC_50_: The concentration required for 50% inhibition
IK_Ca_ channel: Intermediate-conductance Ca^2+^-activated K^+^ channel
K_IR_ channel: Inwardly rectifying K^+^ channel
SEM: Standard error of mean
TRAM-34: 1-((2-chlorophenyl)-(diphenyl)methyl)-1*H*-pyrazole
VGA: Vigabatrin (γ-vinyl-GABA)
